# Intraoperative molecular imaging – it’s all about advancing surgical decision making and improving dexterity^☆^

**DOI:** 10.1016/j.zemedi.2026.01.002

**Published:** 2026-01-20

**Authors:** Fijs W.B. van Leeuwen, Tessa Buckle

**Affiliations:** Interventional Molecular Imaging Laboratory, Leiden University Medical Center, Leiden, the Netherlands

**Keywords:** Molecular imaging, Intraoperative imaging, Robot surgery, Digital surgery

## Abstract

Clinical implementation of intraoperative molecular imaging has accelerated by incorporating molecular image-guided decision making into minimal invasive robotic surgery. The resulting digital surgery concepts combine precise molecular image-guided decision making with increased dexterity, with the aim to achieve value-based medicine. In this review several surgically encountered challenges and the technological innovations the field of intraoperative molecular imaging and molecular image-guided robotics, that include navigation in preoperative imaging roadmaps, dosing, imaging and sensing and performance assessment are discussed. Herein specific focus is placed on physical trade-offs between key issues such as penetration depth, spatial resolution and background signal, as well as integration of hybrid tracers and multimodal strategies.

## Introduction

**Fig. 1:**
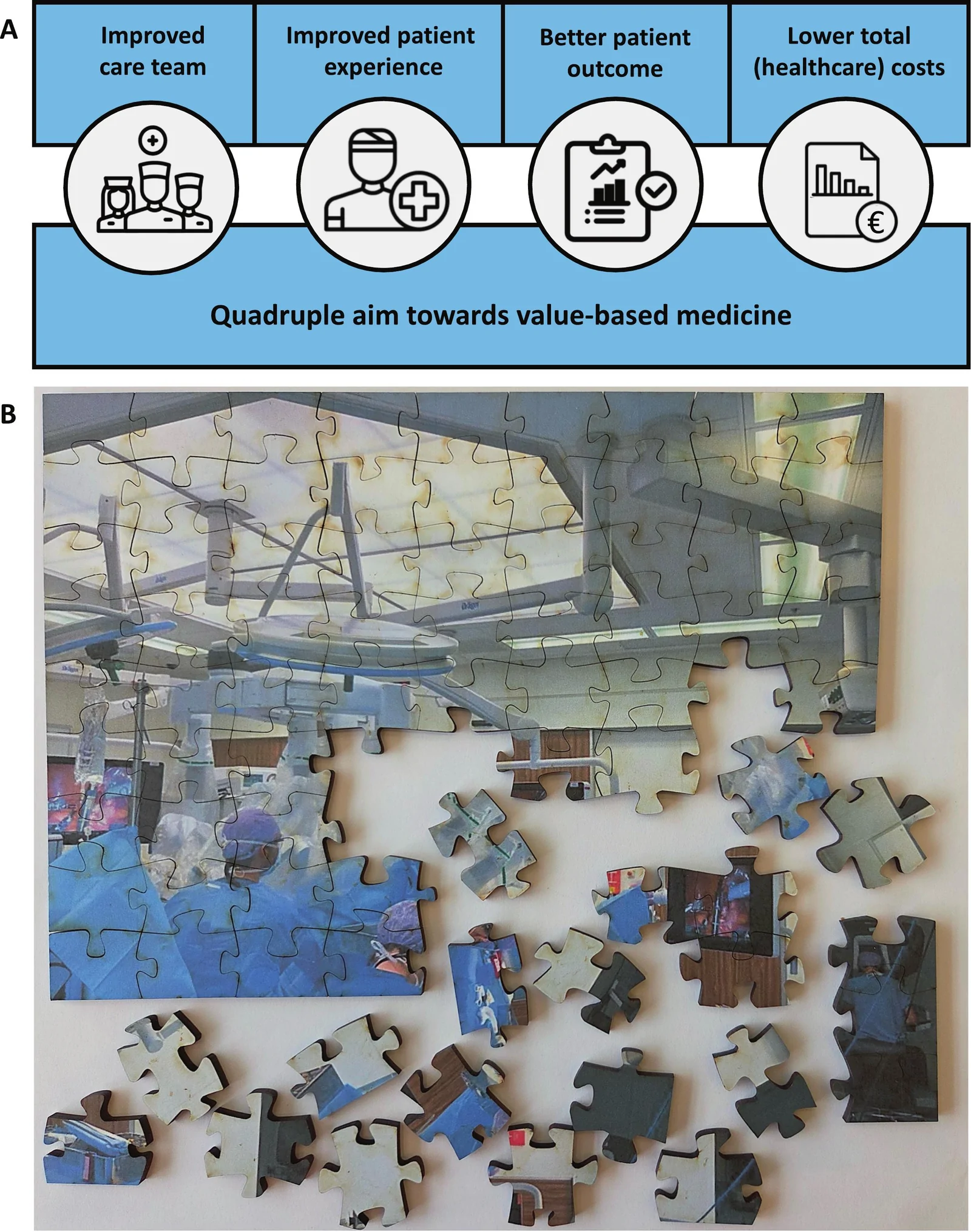
**Molecular image-guided decision making in value-based medicine.** A) quadruple aim of intraoperative molecular imaging strategies towards value-based medicine. B) Puzzle pieces representing the complexity of robotic surgery.

According to a famous quote from Dr. Spencer (1978; “A skilfully performed operation is about 75% decision-making and 25% dexterity” [1]), the act of surgery provides a dynamic environment wherein decision making and dexterity are leading. Ground-breaking engineering solutions in the form of minimally invasive surgical robots endow surgeons with increased dexterity [2,3]. On the other hand, molecular imaging as a discipline promises to improve target perception to a level that allows precise molecular image-guided decision making in situations with complex disease spread and within intricate anatomical landscapes. By making comprehensive medical data-streams accessible for interpretation and further processing, the rise of digital surgery concepts helps tie the two together (Fig. 1) [4,5]. Convergence of the three strategies in intraoperative molecular imaging has the ambition to achieve value-based medicine via a quadruple aim: 1) improved care team- and 2) patient experience, 3) better patient outcome, and 4) lower total (healthcare) costs (Fig. 1A). Here it should be noted that (some) clinical value may already arise when one of these aims has been met, while full appreciation of additional value of an intraoperative molecular imaging technique requires coverage of all four.

Driven by the enhanced dexterity and ergonomics that lie at the surgical robot’s core [6,7], the use of robots during surgeries such as robot-assisted radical prostatectomy (RARP) has been on the rise since its introduction in the early 2000’s [[8], [9], [10]]. RARP is currently applied as standard of care, accounting for 52 - 65% of prostate cancer surgeries [9,11]. The robotic platform has been shown to help improve the surgical experience [12] and minimize recovery times for patients [13]. Furthermore, this technological advancement has been shown to reduce incidence of postoperative erectile [14] and urinary incontinence [15] complications. Precision surgery, however, requires more than improvements in dexterity.

**Fig. 2:**
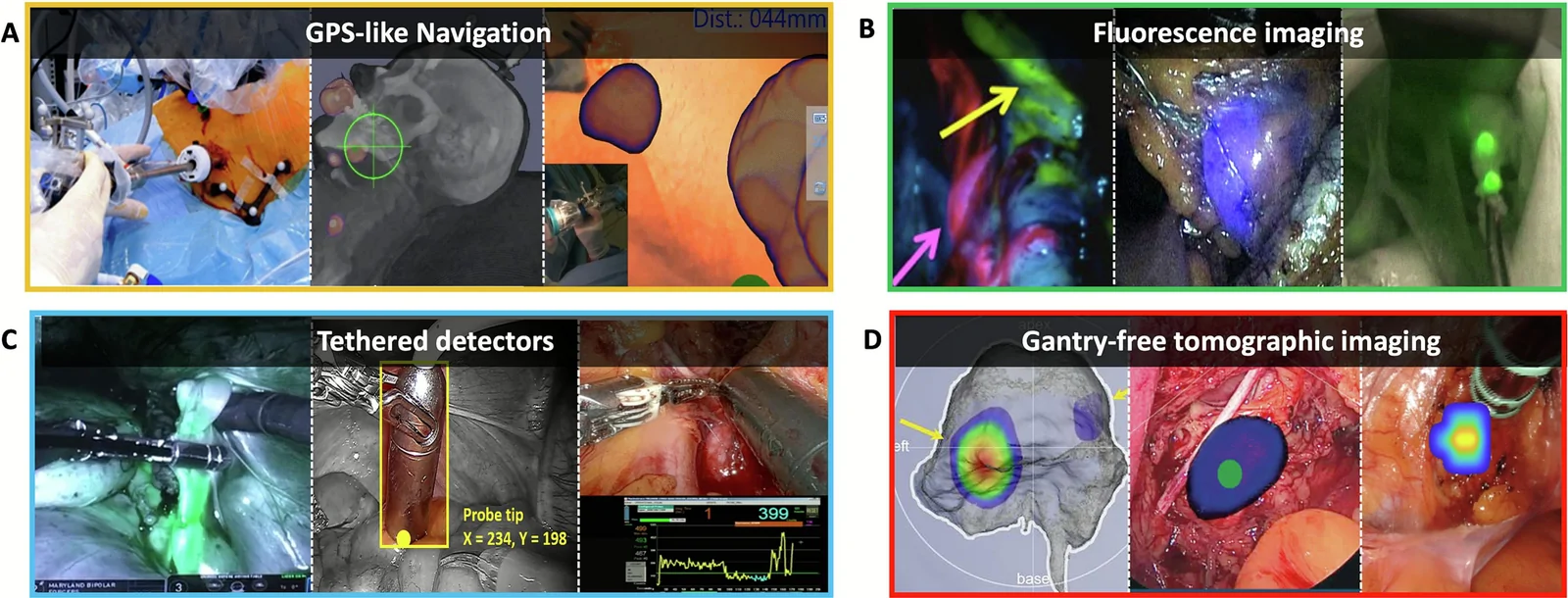
**Intraoperative molecular imaging.** Examples of application of intraoperative molecular imaging: A) GPS-like navigation, showing fiducials placed on the patient (left image), the specified target depicted as the center of a green cross projected onto a 3D SPECT/CT scan (middle image) and distance to target provided within the projected view (right image). B) fluorescence imaging, showing multispectral fluorescence imaging using two separate tracers (fluorescein: yellow arrow, ICG: pink arrow; left image) and visualization of a sentinel node (SN) in blue (middle image) and green (right image) with two different cameras. C) tethered detectors, showing a drop-in gamma probe in fluorescence imaging mode (left image), probe tracking with coordinates of the probe tip (middle image) and integration of the radioactive count rate within the robotic surgical view (right image). D) gantry-free tomographic imaging, showing a light and reflectance (LiDAR) image of an ex vivo prostate specimen with overlay of a freehand SPECT scan (left image), intraoperative freehand SPECT with target projection (middle image) and robotic SPECT obtained using a drop-in tracked drop-in gamma probe (right image).

In a highly interactive environment such as robotic surgery, perception is said to be the root cause behind surgical target identification (decision making) and the curative surgeon-patient interactions that follow thereafter [16]. The intent of molecular imaging to visualize cancer tissue biology aligns with the need to enhance surgical perception to a level that goes well beyond human capacities [2,17]. This clinical need has been the main driver for the pursuit of intraoperative molecular imaging (Fig. 2). Transitioning molecular imaging to the surgical theatre, however, means that advanced imaging must be enabled in a dynamic setting and within a restricted space. Since full blown positron emission tomography (PET) and single photon emission computed tomography (SPECT) gantries obstruct surgery and especially do so in robotic surgery, “small” and highly dexterous imaging solutions are needed to realize intraoperative molecular imaging. In addition, these imaging solutions need to provide guidance at a level that helps expose the cancerous tissue in a minimally invasive manner. Molecular image-guided robotics is an up-and-coming subdiscipline, wherein prostate cancer surgeons are very much taking on the role of early adaptors [2]. The huge scalability of innovations in this field is substantiated by availability of the >10 thousand robotic systems worldwide, the >17 million robotic procedures performed to date, and the >43 thousand peer reviewed scientific papers on surgical robotics [18,19]. When considering all surgical disciplines, there are annually >10 million minimally invasive surgeries that can potentially benefit from intraoperative molecular imaging [20,21]. These include robotic treatment of frequently occurring, but debilitating malignancies, such as renal-, cervical-, hepatobiliary- and colorectal- cancer [22].

To deliver on their promise, the fields of intraoperative molecular imaging and molecular image-guided robotics require innovations that are lifted to a level that they can breakthrough in clinical care. Surgery thereby being akin to a complex puzzle wherein each piece, no matter how small, contributes to the overall performance (Fig. 1B). In line with that, a carefully orchestrated interplay of disruptive but complementary innovations across multiple technical disciplines are required to address the surgical complexity and speed up progress across various stages of the translational pipeline. In this manuscript we put forward several surgically encountered challenges and the technological innovations that can address these issues. Herein the trade-offs in the physical underpinnings of intraoperative molecular imaging that determine its utility are highlighted via discussion of the key issues such as penetration depth, spatial resolution and background signal, as well as integration of hybrid tracers and multimodal strategies.

## Navigation in preoperative imaging roadmaps

From a clinical perspective nuclear medicine (and in particular images generated using gantries that combines PET and SPECT with computed tomography (CT), respectively SPECT/CT and PET/CT gantries) set the standard for molecular imaging [23]. These images are also used to match patients with the most suitable surgical procedure. For example, prostate cancer patients that are node-negative on prostate specific membrane antigen (PSMA) targeted PET/CT but have a >5% Briganti score can benefit from a sentinel node procedure [24]. While patients showing local nodal spread on PSMA-PET/CT (< 3 lesions) are eligible for PSMA-targeted surgery [25]. When the preoperative images show tracer uptake in lesions, they also provide a 3D ‘roadmap’ surgeons can use for navigation purposes (Fig. 2A). These are either based on the surgeon’s ability to cognitively translate what is seen on the ‘roadmap’ to the surgical setting or in a technical navigation setting [4]. The latter is likely to yield the most reproducible results. While the image-to-patient registration required for navigation strategies as effective in rigid tissues, such as those encountered in bone, they quickly lose their precision when tissue movement occur [26,27]. Thus, limiting their value to macroscopic guidance in soft tissue surgery (Fig. 2).

Recent efforts have indicated that for both sentinel node and PSMA-targeted procedures the signal intensities seen in preoperative ‘roadmaps’ correlate to the signal intensities encountered during the surgical act [28,29]. As such, preoperative ‘roadmaps’ can also act as a predictor for the likelihood that a surgeon will benefit from intraoperative molecular imaging.

## Superficial detection vs in-depth intraoperative detection

**Fig. 3:**
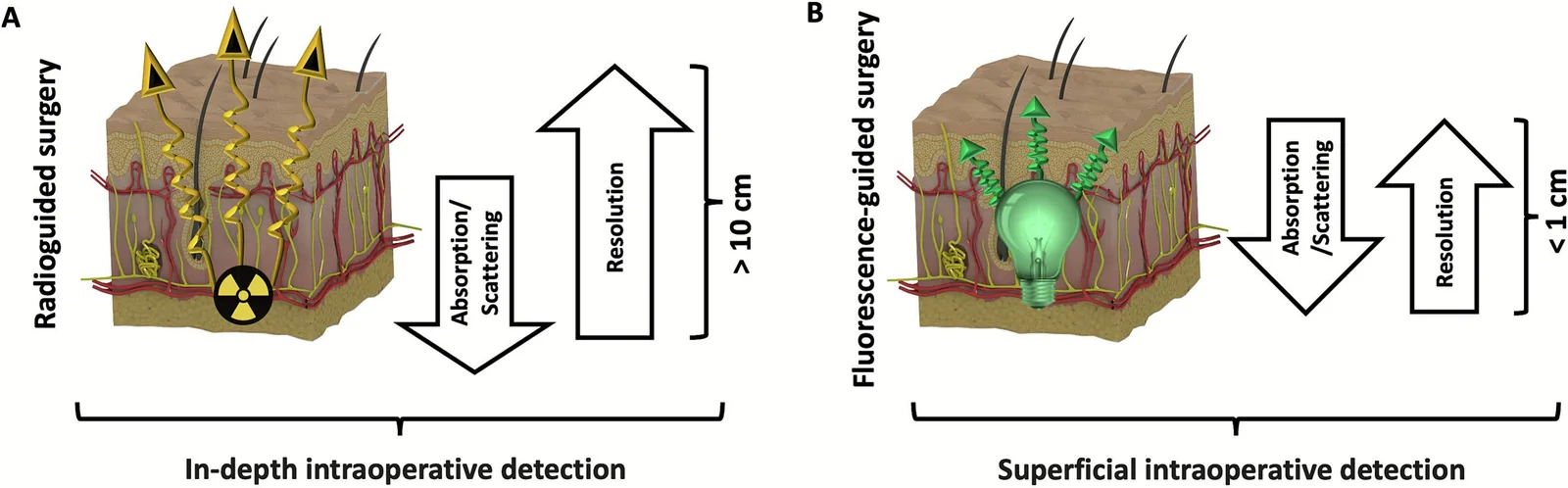
In depth vs. superficial intraoperative detection. Differences in signal penetration and resolution for A) radioguided surgery (suitability for in-depth detection driven by minimal tissue attenuation accompanied by relatively poor spatial resolution) and B) fluorescence-guided surgery (suitability for superficial intraoperative detection driven by high resolution accompanied by relatively low sensitivity). Images adapted from van Leeuwen et al [30].

Surgical procedures typically consist of different phases or steps and the need for image guidance varies greatly between these steps. For example, initial exploration only requires macroscopic insight into the location of a target hidden deep within the patient’s anatomy, while resection of a (primary) cancer requires detailed insight in its margins. From a physics perspective different imaging signatures provide a different level of tissue penetration (Fig. 3, [30]) and with that take a different place in the surgical workflow. For in depth detection, molecular signatures are required that are not, or minimally, attenuated by tissue. The most widely used being gamma emissions 25-300keV (SPECT) or 511keV (PET). While such signatures can also help identify diseased tissues in margins, they suffer from a relatively poor spatial resolution 4-10 mm [31] and are prone to “shine through” of background signal. To get eliminate this effect, molecular signatures with opposing qualities (e.g. that suffer from severe tissue attenuation) can be used [30]. A prime example hereof is fluorescence imaging (Fig. 3B); the promise of real-time imaging of cancer has attracted surgeons to using (near-infrared) fluorescence – an imaging modality that was first-introduced in the robotic arena in 2009 [32]. Fluorescence imaging (Fig. 2B) is suitable for superficial applications, but the relatively low sensitivity severely limits its value during pursuit of lesions hidden below the tissue surface [30]. From a nuclear medicine perspective beta^+^-emitting radioisotopes allow superficial detection using beta^+^-probes [33] or via secondary Cerenkov luminescence [34]. As a consequence of positron range effects (typically on the order of 2-5 mm for many beta^+^-emitters) the achievable spatial resolution in PET is vastly inferior to the sub-micrometer resolution possible with fluorescence imaging.

Strategies that use a cocktail of differently labeled pharmaceuticals can be used to combine in-depth with superficial insights – serving as a best-of-both-worlds approach [35]. All be it that the different pharmacokinetic properties of compounds and differences in dosing may result in “comparing apples and oranges” [36]. Especially when one realizes that radiopharmaceuticals tend to be used at microdosing levels (< 100ug/patient) while fluorescent pharmaceuticals tend to be used at doses that exceed 1mg/patient. Bi-modal or hybrid pharmaceutical that combine two signatures on one molecular platform, and that can be used in a microdosing regimen, help circumvent this shortcoming by ensuring that e.g. the radioactive lesions are also fluorescent [30,35,37]. The most widely used hybrid pharmaceutical in the clinic being indocyanine green-^99m^Tc-nanocolloid [[38], [39], [40], [41], [42]].

## Dosing

**Fig. 4:**
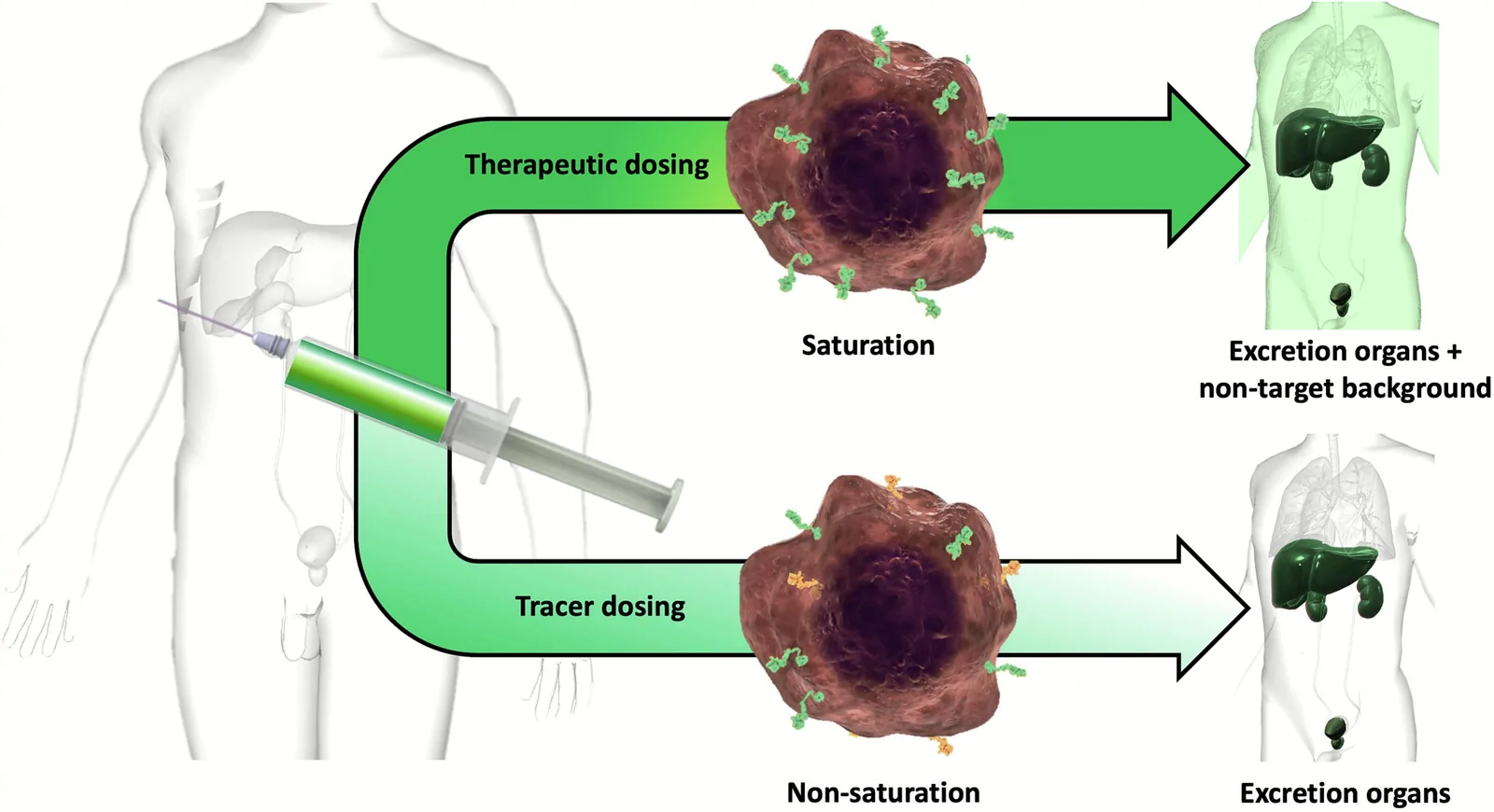
Effect of dosing on signal intensity in receptor-targeted applications. Example of receptor targeting and tracer distribution after intravenous administration of a PSMA-targeted tracer at a A) therapeutic dosing (e.g. >2mg or 1.2 mMol/patient for fluorescent PSMA targeting tracers, [45,46]) and B) tracer dosing regimen (40ug or 30 pMol/patient for PSMA targeted radiotracers, [47,48]). PSMA receptors on membrane of tumor cell (center) depicted in yellow, with tracer-bound receptors depicted in green. Tracer signal represented in green. Cell and anatomical images created using Biodigital.com.

While one would not directly relate dosing of pharmaceuticals to imaging physics, the amount of agent injected into the patient and the route of administration greatly influence the signal intensities obtained in the target as well as background tissues [36]. Common sentinel node (SN) procedures relying on local injection show accumulation of 1-10% of the injected dose [43], with the remaining activity located at the site of injection often serving as background [44]. When pharmaceuticals are administered intravenously, everything changes. Not only does the lesion accumulation have to be based on active uptake, but it also needs to compete with background uptake throughout the body and bodily clearance mechanisms (renal and/or hepatic, Fig. 4). Recent studies that implemented fluorescent PSMA-targeting pharmaceuticals (e.g., IS-002 and OTL78) showed that therapeutic dosing (>2mg or 1.2 mMol/patient) did not allow reliable PSMA-receptor targeting [45,46].

Most targeted pharmaceuticals exhibit a nanomolar affinity in the form of IC_50_ of K_d_ value, these values tend to represent the concentration at which 50% of the PSMA receptors are saturated in vitro. While it is impossible to directly translate such values to an in vivo setting, they indicate that a substantial number of receptors should be available on the cell membrane to allow all the administered pharmaceutical molecules to bind to a receptor. In other words, high dosing regimens that correspond to therapeutic dosing likely constitutes as overdosing. The cost being that overdosing increases background signals (Fig. 4A) and promotes false positive results, without improving the visualization of the e.g. PSMA-expressing targets. In that sense, higher dosing of fluorescent pharmaceuticals does not improve the detection of lesions hidden at a depth of > 0.5-1cm in the tissue. Nor does it help overcome the related false positive findings [36,45,46]. Interestingly, a radiopharmaceutical for the exact same target (^99m^Tc-PSMA-I&S) used under a microdosing regimen (40ug or 30 pMol/patient, [47,48]) does allow accurate lesion detection (Fig. 4B) [25,49,50].

When comparing SN procedures with receptor targeted-procedures an eight-fold drop in signal intensity in a lesion and a two-fold decrease in the signal-to-background ratio was observed [51]. These findings suggests that receptor-targeted approaches are inherently more prone to false negative and false positive findings in terms of surgeons using the signatures to restrict the resection to tissue that illuminates intraoperatively.

## Imaging vs sensing

Human interactions rely on a vast array of sensory experiences, with two prominent ones that are indispensable for surgery being vision and touch. During surgery these sensory experiences can be converted to imaging and probe-based tracing, which are both being implement on a large scale in the clinical routine. For intraoperative imaging fluorescence cameras dominate clinical applications. This is supported by availability of (near-infrared) fluorescence camera from all major surgical equipment vendors, and full integration of a fluorescence endoscope in robotics platforms such as the da Vinci robot [52]. For a surgeon the interpretation of real-time visualization of fluorescence is seemingly very intuitive. For gamma-emissions there are several portable gamma-camera solutions available, but these provide static rather than dynamic images [53]. Context for the observed radioactive hotspots is provided by merging with white light images [54]. On the other end gamma-, beta^+^-, and fluorescent-probes are being used to trace signals via a counts/s readout, which corresponds to ‘touch’ [2]. Interpretation of these readouts can be challenging, especially when signals are low and background is high. To make molecular sensing robot-compatible generation 1 and 2 (Gen 1&2) drop-in gamma-sensor technologies have been developed (Fig. 2C, [[55], [56], [57], [58]]). Since then there a drop-in beta^+^ probe has also made its way into clinical trials [33] and fluorescence sensing has shown promise on surgical specimens [59].

**Fig. 5:**
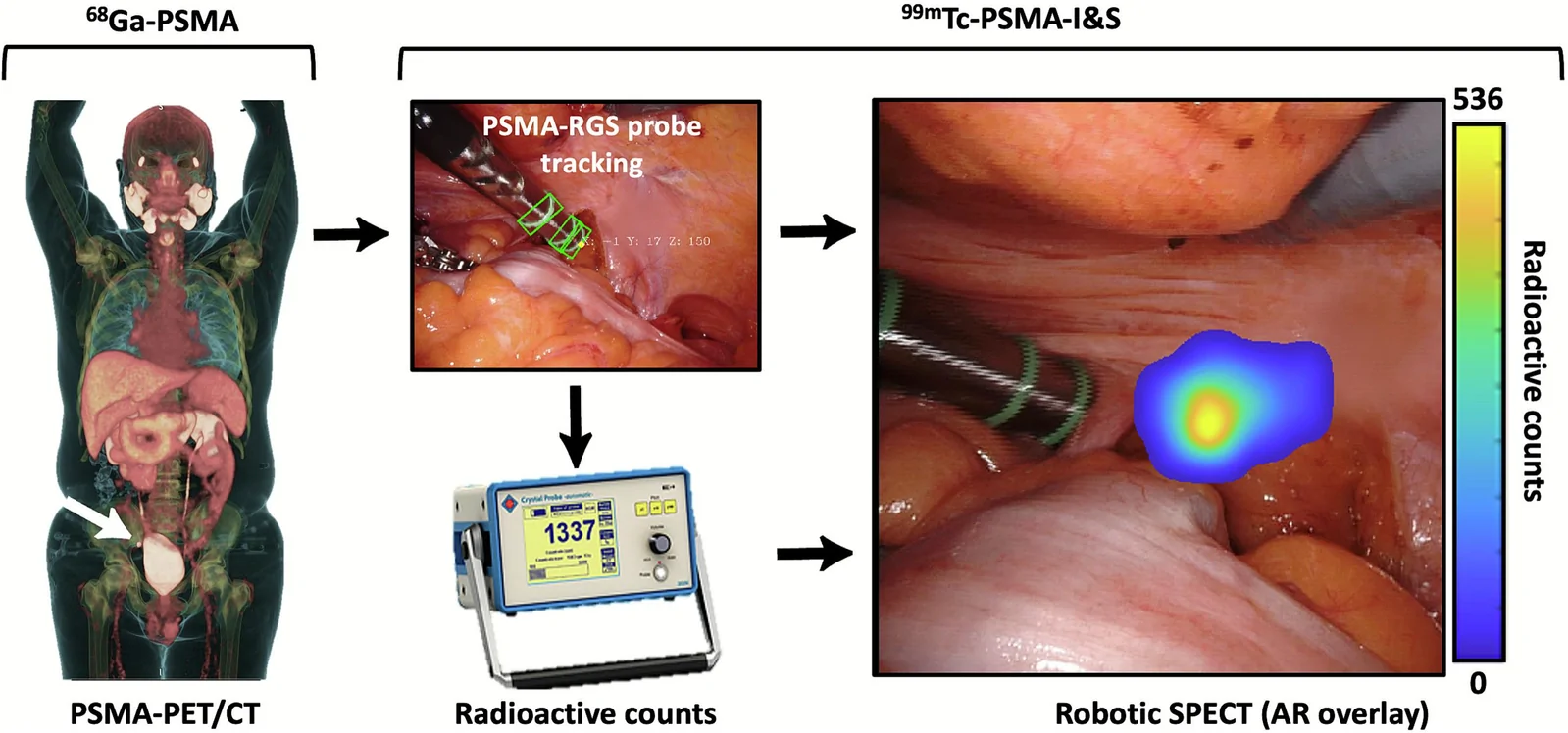
**Gantry-free robotic SPECT**. Workflow of the creation of intraoperative gantry-free robotic SPECT with surgical planning based on PSMA-PET/CT (using e.g. ^68^Ga-PSMA-11) in the same patient. Intraoperative tracking of a tethered drop-in gamma probe combined with quantitative radioactive count rates (after administration of ^99m^Tc-PSMA-I&S) form the basis of an intraoperative SPECT scan. The resulting gantry-free robotic SPECT scan enables depiction of the radioactive target within the robotic view by means of an artificial reality (AR) overlay). Scaling represents the count-rate within the robotic SPECT overlay.

The unique concept of declipseSPECT enables surgeons to transfer their hand-held gamma-probe interactions with the patient into 3D images that can be overlayed on the system integrated 2D camera vision [60] or even on 2D endoscopic vision [61]. This process is facilitated by optical tracking of reference fiducials placed on the patient (Fig. 2A) and gamma probe which essentially provide a gantry-free SPECT solution that helps make in depth 3D imaging possible in a surgical setting (Fig. 2D). The gantry-free imaging paradigm has also been expanded towards beta^+^- [62], MRI- [63] and fluorescent-signatures [64]. When combined with light detection and ranging (LiDAR) the 3D images can be placed in 3D anatomical context (Fig. 2D) [65]. Recently roboticSPECT was introduced as an extension of the current two-tracer approach that is used for PSMA-guided surgery [66]. Herein ^68^Ga-PSMA PET is employed for patient selection and intraoperative radioguidance is facilitated by ^99m^Tc-PSMA-I&S (Fig. 5) [58]. The longer half-life of the ^99m^Tc isotope makes it more suitable for intraoperative detection purposes. Acquisition of roboticSPECT requires a drop-in probe and video-based tracking of dedicated fiducials that provides the means to create a gantry free SPECT scan during the surgical intervention.

## Performance assessment

Real-world evidence of impact is critical for defining the value of new surgical precision enhancing technologies [67]. Such evidence allows data-driven quality control and proficiency standardization against relevant endpoints. In that sense it should be standard practice to evaluate new technologies against clinical endpoints such as correlating surgical findings to preoperative imaging roadmaps, pathology (“golden” reference standard), complication rates, recurrence, and long-term survival. Unfortunately, most clinical studies that feature novel technologies only show first-in-human proof-of-concept data. While first-in-human data is exciting and helps moves the field forward, ultimately a technology needs to address the quadruple aim (Fig. 1). With regards to patient outcome, the main parameter is the improvement of clinical endpoints such as reduced complications, reduced recurrence rates and increased overall survival. Very few studies address this with some exceptions being SN [68], 5-ALA [69], hybrid SN prostate [70] and PSMA-targeted prostate surgery [71]. While the body of evidence is growing within the research field of intraoperative molecular imaging, it is still not routine to link technological innovations to patient benefit.

Another important aspect is the effect that innovative perception enhancements have on the proficiency of the surgical staff. In general, qualitative assessments (opinions or questionnaires) of surgical experiences are being provided at most. In some instances, Delphi consensus has been conducted among experts [72,73]. In the era of digital surgery, kinematics help provide an objective and quantitative readout i) for the effect that particular sensor designs have on the surgical dexterity [74,75], ii) that helps define how molecular insights impact surgical decision making [51,76], and iii) automated classification of surgical phases [77]. When directly related to clinical endpoints these insights support the establishment of procedural proficiency.

Lastly, patient experience is a challenging point to address. As the internet makes knowledge accessible for patients and their families, it becomes increasingly common to see patients request e.g., robotic or fluorescence-guided surgery. In that sense, the feeling that they are being treated in line with the current technological state-of-the-art enhances their experience. The same is true for reduced hospital stay. But ultimately it is safe to assume that patients experience is very dependent on the surgical outcome in terms of surgical complications and side-effects as well as disease recurrence and the need for impactful follow-up therapies. A decrease of total (healthcare) costs can only be achieved when all contributing features are aligned and when technologies are being implemented on a large scale.

## Conclusion

The clinical implementation of intraoperative molecular imaging is evolving rapidly. While great strides have already been made regarding the use of hybrid tracers and multimodal technologies, open research challenges include miniaturization, real-time quantification and AI-based data fusion. Currently, gathered expertise is maturing insights in how clinical needs can be converted into technical solutions, and how these can best be evaluated to align to the quadruple aim of improved care team, patient experience, increased patient outcome, and lower total (healthcare) costs.

## CRediT authorship contribution statement

**Fijs W.B. van Leeuwen:** Writing – review & editing, Writing – original draft, Conceptualization. **Tessa Buckle:** Writing – review & editing, Writing – original draft, Conceptualization.

## Funding

This research was supported by a Dutch Research Council NWO KICH grant (KICH1.ST03.21.030).

## Declaration of competing interest

The authors declare that they have no known competing financial interests or personal relationships that could have appeared to influence the work reported in this paper.
